# Fundamental Aspects of the Development of a Model of an Integrated Health Care System for the Prevention of Iron Deficiency Anemia among Adolescent Girls: A Qualitative Study

**DOI:** 10.3390/ijerph192113811

**Published:** 2022-10-24

**Authors:** Puspa Sari, Dewi Marhaeni Diah Herawati, Meita Dhamayanti, Dany Hilmanto

**Affiliations:** 1 Doctoral Study Program, Faculty of Medicine, Universitas Padjadjaran, Bandung 45363, Indonesia; 2 Department of Public Health, Faculty of Medicine, Universitas Padjadjaran, Bandung 45363, Indonesia; 3 Department of Child Health, Hasan Sadikin Hospital, Faculty of Medicine, Universitas Padjadjaran, Bandung 45363, Indonesia

**Keywords:** iron deficiency anemia, adolescent girls, health care system

## Abstract

Iron deficiency anemia (IDA) in adolescent girls is a problem that has not been resolved. This study aimed to explore the critical aspects of an integrated health care system model for preventing IDA in adolescent girls in a rural area of Indonesia. This qualitative research employed a grounded theory approach in order to build a substantive theory. This study used in-depth interviews with adolescents, parents, teachers, health workers, and persons in charge of adolescent programs at the health office, education office, and ministry of religion. Purposive sampling was performed until data saturation was achieved. Codes, categories, and themes were generated through thematic data analysis to develop a substantive theory. Data analysis was performed using MAXQDA 2022 software. A total of 41 people participated in this study. This investigation generated twenty-two categories and seven themes. These themes relate to policymaker commitments, stakeholder governance, quality, adolescents’ lifestyles, adolescents’ self-factors, adolescents’ access to health services, and social support. The themes identified become fundamental aspects of the integrated health care system model for preventing IDA in adolescent girls. The model of the integrated health care system consists of several essential points, which include awareness and efforts from policymakers and adolescent girls, supported by parents, teachers, and the community.

## 1. Introduction

Iron deficiency anemia (IDA) is a global health problem in developed and developing countries. IDA is one of the contributing factors to the global burden of disease [1,2,3,4]. According to the World Health Organization (WHO), the problem of IDA in Indonesia can be classified as moderate [5]. The cut-off hemoglobin level for diagnosing anemia in adolescents is 12 g/dL [6,7]. IDA in Southeast Asia, including Indonesia, is caused by a lack of nutritional intake, especially iron [5,8,9,10]. IDA occurs at all stages of life, but is more common in women of childbearing age, pregnant women, toddlers, children, and adolescents [11]. According to the 2013 Indonesian Basic Health Research, around 22.7% of adolescent girls experience IDA [12].

According to the WHO, people who are 10–19 years of age are classified as adolescents [13]. Adolescence is a transitional period from childhood to adulthood. There is an increase in the physiological need for iron due to accelerated growth, the expansion of body mass, increased blood volume, and blood loss due to menstruation [2,14,15]. In addition, the causes of IDA are parasitic infections such as hookworms, lack of knowledge about IDA prevention, parental education, and socioeconomic status [3,14,16,17]. Other contributing factors include physical activity, a lack of sanitation, personal hygiene, the number of family members, and the provision of food by parents [14,18,19].

IDA in adolescent girls can result in stunting, mood swings, decreased concentration and cognitive abilities, reduced productivity and immune system, and reproductive health disorders [5,20,21]. IDA in pregnant adolescents can result in delivery problems such as stillbirth, low birth weight (LBW), prematurity, and decreased iron status in newborns [2]. Furthermore, the WHO states that IDA is one of the factors that can cause maternal death [5,22].

Several programs for preventing IDA have been implemented in Indonesia. The efforts made include the provision of iron supplements containing 60 mg of elemental iron and 400 mcg of folic acid, as well as health education regarding balanced nutrition, with a focus on the need for nutritional improvement [5,9,23]. Iron supplements are provided in several places, such as schools, namely School Health Units (in Indonesia, these are called UKS), community health centers (in Indonesia, these are called Puskesmas), through adolescent health care (in Indonesia, this is called PKPR), and adolescent integrated services in the community (in Indonesia, this is called Posyandu) [24]. However, there are still many obstacles and gaps in the program to prevent IDA in adolescents [9]. The main obstacle concerns the absence of complete data on the number of IDA cases, making it difficult for policymakers to focus on the problem. Additionally, adolescents continue to reject iron supplements, which reduces their coverage. Only a few countries have comprehensive programs that have achieved adequate coverage [5]. Furthermore, the attention of policymakers and those in charge of programs is important. However, efforts to avoid IDA have thus far only been concentrated on a small number of adolescents who are at risk [23].

A model of an integrated health care system is needed to prevent IDA. There is no clear definition of integrated care. However, in general, it can be interpreted as a practice that seeks to improve patients’ health and their experiences of the health care delivery system [25]. Many integrated health care system models have been developed, such as the Chronic Care Model for chronic diseases and models for integrated health services [26,27,28]. However, there is no specific model for IDA. This study aims to explore the fundamental aspects of building an integrated health care system model to prevent IDA in adolescent girls.

## 2. Materials and Methods

### 2.1. Study Design

This research is qualitative research based on a grounded theory (GT) approach using the constructivism paradigm [29,30,31]. GT is strict but flexible, starting with openly gathering information and then systematically analyzing the inductive data derived from codes, categories, and themes, leading to theory development [30,31]. The theory produced in this study is a model of an integrated health care system for the prevention of IDA in adolescent girls.

### 2.2. Data Collection

The Soreang sub-district is a rural area in Indonesia, and served as the site of this study’s fieldwork. This area is a rural area with a distance of 22.5 km from the province’s center. Our study took the data from public and private high schools (SMA), vocational high schools (SMK), and Madrasah Aliyah (MA) between October and November 2020. Participants in the study were provided with information and signed a consent form. In-depth interviews were conducted face-to-face to explore phenomena or constructs. Interviews were conducted until the data were saturated, which means that no additional data could be found. The research questions were “What forms of iron deficiency anemia prevention programs exist?”; “What are the barriers to iron deficiency anemia prevention programs?”; “What types of health services exist for adolescent girls to prevent iron deficiency anemia?”. Interviews were held for 30–45 min. Data were recorded by audio recording and written notes as a research instrument. Interviews with adolescent girls and their parents were conducted in their respective homes. Meanwhile, interviews with teachers, health workers, and policymakers were conducted in their workplaces.

Sampling was performed using the purposive sampling technique to obtain information from participants who provided or received health services aimed at the prevention of IDA. The participants in this study were adolescent girls, parents, teachers, health workers, and individuals in charge of adolescent programs at the health office, education office, and the ministry of religion. The number of samples taken in this study was determined on the basis of theoretical sampling, whereby samples were taken until data saturation was reached [29,32]. The inclusion criterion for an adolescent girl was late adolescence. The consideration in selecting late adolescence was that most adolescents marry after completing senior secondary education in rural areas. It is essential to prevent IDA from an early age in order to provide protection during pregnancy.

This study was conducted following the guidelines of the Declaration of Helsinki. It was approved by the Ethics Committee of the Faculty of Medicine, Padjadjaran University, Bandung, with the code number 756/UN6.KEP/EC/2020. Informed consent was received from all subjects before the study.

### 2.3. Data Analysis

The steps taken to perform qualitative data analysis were as follows: (1) prepare the data for analysis by transcribing the recordings into a narrative, as well as the notes that were made; (2) perform a data reduction process, namely reading the narrative results several times, selecting data, and eliminating unnecessary words; (3) read the results of the narrative, producing codes in the form of words or phrases from the results of the narration, referred to as the coding process; this coding process can be performed manually using a computer program, and the coding results were divided into categories; (4) create a theme from the category results [30]. Data analysis was performed using MAXQDA 2022 software.

Our study used a trustworthiness component consisting of four criteria—credibility, dependability, confirmability, and transferability—to achieve quality or rigor in this study [30,31]. Credibility determines whether the findings represent the original views of the participants. Researchers facilitated transferability assessments by potential users on the basis of quick descriptions. All were supported by data obtained from the research participants. Confirmability is concerned with establishing whether the interpretations of findings are derived from the data [30,31].

## 3. Results

Informants who participated in the study were adolescent girls, teachers, parents, health workers from the public health center, and individuals in charge of adolescent programs from the health office, education office, and the ministry of religion (Table 1). The adolescent girls who participated in this study came from 16 schools. Most teachers who participated held bachelor’s degrees and served as the person in charge of the adolescent program at the school (Table 1).

The health workers in this study came from two public health centers. The education level of the health workers was a bachelor’s degree in public health education and nutrition. In addition, our research involved those in charge of adolescent programs at the health, education, and religious ministries, with the majority of education levels being diploma, bachelor’s, and master’s degrees (Table 1).

After combining aspects on the basis of the informants’ perspectives, 41 transcriptions, 51 codings, 22 categories, and seven themes were obtained, which became fundamental aspects in the integrated health care system model of IDA in adolescent girls (Table 2). The aspects found on the basis of the analysis in the first stage then became a substantive theory for an integrated health care system for IDA, namely policymaker commitment, stakeholder governance, quality of health services and iron supplements, adolescent lifestyle, quality, adolescent self-factors, adolescent access to health services, and social support. 

### 3.1. Theme 1: The Commitment of Related Institutions

#### 3.1.1. Adolescent Counseling Room

One effort to prevent IDA in adolescent girls is through counseling; in this case, a particular room is needed to convey information. However, the participants stated that there are no rooms—for example, in the school health unit (UKS) or in the space for the adolescent health care program (PKPR) at the community health center.

*There were no rooms for counseling such as UKS; health education is carried out outside the classroom, even though we need a special place for adolescent counseling* (Interviewee 1).

#### 3.1.2. Availability of Infrastructure

In this study, respondents revealed that there had never been a hemoglobin test. Other respondents stated that the availability of tools, especially hemoglobin examination tools, was lacking, so hemoglobin examination was not carried out.

*Facilities are less limited, especially hemoglobin sticks, so blood examination to diagnose anemia are limited* (Interviewee 2).

#### 3.1.3. Availability of Financial Support

Adolescents declared that health programs are still hampered by available funds, such as funds for adolescent health education, depending on the availability of funds.

*Health education for adolescents already exists from the puskesmas. Still, it depends on each puskesmas, because this activity is related to funds, so it depends on the ability of the puskesmas* (Interviewee 3).

#### 3.1.4. Quality of Health Workers

The ability to record the outcomes of care and communication skills are two aspects of health professionals’ quality that were examined in this study. Communication skills are necessary for health professionals to effectively and efficiently deliver information to adolescents in a way that they can understand. The participants stated that they did not record the services.

*Our recording has not been documented, only in the form of an oral report* (Interviewee 2).

#### 3.1.5. Health Worker Behavior

It is necessary for health workers to take responsibility and perform follow-ups in order to prevent IDA. Participants revealed that health workers only entrusted iron supplements to teachers, and often no further monitoring was carried out.

*The teacher gives the iron supplements, and the health worker only keeps the tablet; besides that, counseling is only one time, and there is no further examination* (Interviewee 1).

#### 3.1.6. Availability of Iron Deficiency Anemia Health Workers

Participants stated that to provide adolescents with better care for the prevention of IDA, it is essential to have specialized healthcare professionals who deal with this condition. The jobs in the IDA prevention program are not ideal, according to this study’s assertion that health workers must perform a great number of tasks.

*There are too many adolescents, insufficient human resources, insufficient time, and other tasks for health professionals* (Interviewee 3).

### 3.2. Theme 2: Governance

#### 3.2.1. Evaluation Monitoring

One of the participants revealed that the relevant agencies must take an active part in monitoring adolescent programs, since doing so is crucial. Given that there is no dedicated staff focusing on health programs, the monitoring of adolescent health programs by the education office and the ministry of religion was not optimized as part of this study.

*Specifically, there is no monitoring, and we are waiting for reports from the school* (Interviewee 4).

#### 3.2.2. Assignment

Participants said that to ensure continuity, there should be assignments and handovers of tasks from old to new employees, specifically regarding the iron deficiency anemia prevention program. Nevertheless, people in charge of adolescents are unaware of the iron deficiency anemia prevention health service program.

*There is no task to develop UKS, and I am special for students who previously had no technical guidance or socialization* (Interviewee 4).

#### 3.2.3. Coordination

To date, the health service has been the only focus in efforts to deal with iron deficiency anemia. The success of efforts to reduce iron deficiency anemia requires coordination among all relevant agencies, such as the education office and the ministry of religion.

*The health office wants to coordinate more with the education office and the ministry of religion to support each other and to form quality human resources because we will not be able to intervene in schools if there is no policyholder* (Interviewee 3).

#### 3.2.4. Standard Operating Procedures (SOPs)

The participants stated that there was no similarity or even distribution of iron supplements. The informant also said that there was no examination before the distribution of iron supplements. In Indonesia, the distribution of iron supplements is carried out using a “blanket approach”, meaning that all adolescents are given iron supplements without first undertaking a hemoglobin test.^27^ However, it is necessary to have a history and physical examination to ensure that the adolescents should be given iron supplements in the first place. In some instances, such as in cases of infection, adolescents should not be given iron supplements. Some of these problems are related to the availability of standard operating procedures (SOPs).

*If there is counseling, there should also be an examination so that we know whether our child is anemic or not* (Interviewee 5).

### 3.3. Theme 3: Adolescents’ Lifestyles

#### 3.3.1. Knowledge

Some adolescents said that they did not know the general description of iron deficiency anemia—for example, its definition, prevention, signs, and symptoms.

*In terms of understanding, they do not know and do not think that iron deficiency anemia is not a disorder; it is normal. They are sometimes reversed between low blood pressure and iron deficiency anemia* (Interviewee 3).

#### 3.3.2. Healthy Lifestyle

Iron deficiency anemia can be successfully avoided by leading a healthy lifestyle. Additionally, adolescents stated that they had not adopted balanced diet plans, and they continued to have a negative habit of snacking.

*I rarely eat and do not like vegetables* (Interviewee 6).

### 3.4. Theme 4: Quality

#### 3.4.1. Quality Services

The completeness of the data is a factor contributing to the program’s performance; however, in this study, several respondents stated that the number of schools that had the reports or data was still limited.

*There are no reports, but coincidentally, we saw complete health books and how to take iron supplements at one school we visited* (Interviewee 7).

#### 3.4.2. Quality of Iron Supplements

The quality of the iron supplements was regarded by the informants to be related to taste.

*We believe that the taste of iron supplements is unpleasant and makes people queasy* (Interviewee 2).

### 3.5. Theme 5: Adolescent Self-Factors

#### 3.5.1. Trust

Some adolescents revealed that taking iron supplements can cause high blood pressure.

*Students are worried when taking tablets to add blood to high blood pressure* (Interviewee 1).

#### 3.5.2. Motivation

Some adolescents feel excited to receive counseling regarding iron deficiency anemia; in addition, their compliance with respect to taking iron supplements can be seen in their enthusiasm to receive them. However, some adolescents still do not realize the importance of information regarding iron deficiency anemia.

*It seems that adolescent girls do not care about their health, particularly iron deficiency anemia, seems to be unimportant to them* (Interviewee 1).

### 3.6. Theme 6: Access to Health Services

#### 3.6.1. Access to Health Information

Respondents claimed that little was known about iron deficiency anemia. Before being given iron supplements, adolescents should be given health education regarding the definition of iron deficiency anemia and the efforts that can be made to prevent it.

*The service is lacking; ideally, counseling should be given before being given iron tablets* (Interviewee 1).

#### 3.6.2. Access to Community Health Services

Some respondents lived far from the puskesmas; this is one of the obstacles of health services in attempting to achieve iron deficiency anemia prevention.

*It looks like there is a puskesmas in the community, but the puskesmas is far away, a round trip costs 20 thousand, so we don’t know at the puskesmas there are any programs* (Interviewee 5).

#### 3.6.3. Access to Iron Supplements

Several adolescents had not received iron supplements, even though the government launched this program for all adolescents in Indonesia.

*There has never been a distribution of iron supplements here* (Interviewee 1).

### 3.7. Theme 7: Social Support

#### 3.7.1. Parent/Family Support

The role of parents is critical in efforts to reduce iron deficiency anemia. The support that parents/families can give includes the provision of food for adolescents. Another critical factor is the family’s understanding of iron deficiency anemia and its prevention.

*Parents are not given medicine or supplements, they give herbal medicine* (Interviewee 6).

#### 3.7.2. Teacher/School Support

Teachers can carry out iron deficiency anemia interventions through school programs by providing information and time for adolescent health programs.

*They do not give iron supplement because they are busy with their jobs* (Interviewee 2).

#### 3.7.3. Community Support

One of the forms of support that the community can give to assist in the prevention of iron deficiency anemia is the provision of posyandu for adolescents. However, to date, there has been no posyandu for adolescents in this region.

*So far, the community is good; it supports activities, gathers adolescents, and actively prepares the place, but there are no adolescents posyandu yet* (Interviewee 2).

## 4. Discussion

**Figure 1:**
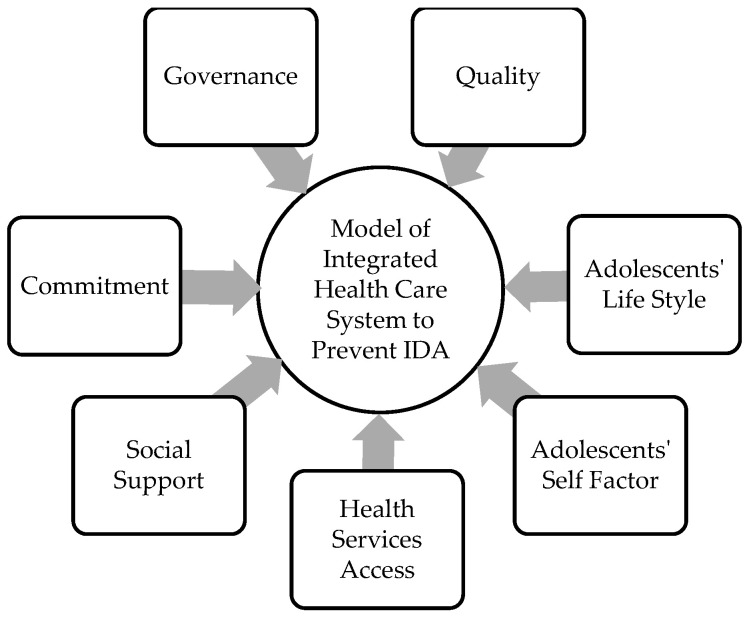
Model of integrated health care system to prevent IDA.

There are some similarities between aspects of the CCM model and those found in this study. However, this study found differences between the substantive theory and the CCM model. Moreover, the theoretical proposition of this study is a model of an integrated health care system for the prevention of IDA, consisting of policymaker commitment, stakeholder governance, quality, adolescent lifestyles, quality, adolescent self-factors, adolescent access to health services, and social support (Figure 1). The integrated health care system model in this study aims to prevent IDA in adolescent girls.

In this study, commitment is related to the accessibility of counseling for adolescents and the availability of health service rooms, as well as the availability of facilities, funding, and the attitudes of health workers and paramedical personnel in IDA programs. In line with the research of Darmawati et al., the management of iron deficiency anemia must be supported by facilities and financial support [33].

A facility that must be available is UKS, which functions as a place for health promotion and prevention, including the prevention of IDA and the fostering of a healthy school environment. However, of the 16 schools studied, only one UKS was running as it should, and there were even schools that did not have a UKS. One of the obstacles to UKS activities is the absence of UKS teachers. Apart from UKS, the availability of the Adolescent Care Health Service Program (PKPR) is also limited. This has an impact on the availability of counseling services for adolescents.

Limited facilities and infrastructure are also obstacles for health workers in conveying health information [33]. Health facilities are an important factor in handling IDA in adolescents. These data are supported by research conducted by Darmawati in Aceh Besar, suggesting that health care facilities are an important factor that must be considered in the management of iron deficiency anemia prevention [33]. Hemoglobin examination facilities for adolescents are still lacking, so hemoglobin examination does not accompany the provision of iron supplements. This is supported by research conducted by Widyawati et al., reporting that facilities supporting iron supplementation programs are still lacking [34,35]. Facilities are not the only problem; another is the manner in which iron supplements are distributed. The availability of iron supplements to adolescents in schools is better than that for pregnant women, toddlers, and children. However, the cost of supplementation programs aimed at covering a large proportion of the large school-age population is relatively high [36].

Another factor in health services related to IDA is that explanations from health workers are still unclear; this is related to the communication skills of health workers. In addition, after iron administration, supervision and responsibility are still lacking [36]. Moreover, documentation or reports on the provision of iron supplements are still an obstacle; for example, there are no reports on adolescents receiving iron supplements, or data on the incidence of IDA. This problem is related to the competence of health workers, which needs improvement. Training is needed to improve the competence of health workers when providing health services for the prevention of IDA [33]. However, some aspects are satisfactory, such as the accuracy of the officers. These results are similar to those reported in a study conducted by Louzado in Peru; the problem of lacking and/or unclear counseling is still visible in the community [36,37]. Counseling is still limited, perhaps due to limited human resources.

Human resources, in terms of both the limited number of health workers and those in charge of the IDA program, became an aspect of this study. This is in line with research conducted at the Puskesmas in Yogyakarta, which revealed that the lack of human resources was causing an overload of work. In addition, health workers feel overwhelmed at work because, as the data show, the number of health workers is not evenly distributed [38]. It is necessary to have health workers who handle IDA prevention programs in adolescent girls focusing on this problem.

The governance in this research referred to monitoring, assignment, coordination, and standard operating procedures (SOP). Governance is an important factor that must be considered, because iron deficiency anemia programs are often not well targeted [34]. Research conducted in Nepal and Pakistan revealed that programs and policies need to be clear and capable of achieving hard-to-reach targets [39]. Other studies have reported that unsystematic protocols can be a problem in iron deficiency anemia programs [37]. Furthermore, the administration of iron supplements requires clear SOPs, so that there is no difference in the amount and method of taking iron supplements. This is in line with the research conducted by Darmawati et al. on pregnant women in Aceh-Indonesia; there is no precise dose for iron supplements, so the amount of iron given to mothers varies [33]. The implementation of efforts to prevent iron deficiency anemia requires continuous monitoring and evaluation [1]. One of the shortcomings of the governance system is inadequate supervision [36].

This study also found no division of tasks or responsibilities for adolescent health programs. However, staff in charge of adolescent health programs are highly necessary. Moreover, there is no division of tasks and responsibilities among related agencies, including in the distribution of funds. For example, the responsibility for printing student health report books, checking hemoglobin, and training UKS teachers, which requires sufficient funds, is left to the health office alone. Therefore, IDA control strategies must involve input from health and non-health sectors [1,9]. Stakeholder cooperation is also an important part of CCM theory [40]. Coordination efforts should be made between stakeholders in order to prioritize the nutritional needs of adolescents [9,25].

Aspects of adolescents’ lifestyles related to IDA were also found in this study. Conducting nutrition education and changing adolescents’ eating habits are the best ways to prevent IDA [41]. In this study, adolescents were found to still have the habit of buying unhealthy food or snacks. This habit arises because there is still a lack of knowledge among adolescents about balanced diets, including the prevention of iron deficiency anemia through the development of healthy eating habits. With respect to health behavior, especially food intake, adolescent fruit and vegetable consumption is still low. This result is in line with research by Garcia-Casal et al. Therefore, nutrition education is needed to increase knowledge about iron deficiency anemia and its prevention [42]. Research by Fentie et al. stated that adolescents still do not know about IDA [43]. Similar research was conducted in East Java and East Nusa Tenggara, indicating that adolescent girls have low levels of knowledge, attitudes, practices, and awareness with respect to iron deficiency anemia and WIFAS [44]. This outcome is in line with research in India reporting that adolescents do not know about iron deficiency anemia and its prevention [45]. However, this study is not in line with the study results reported by Yidana et al., who reported that adolescent iron folic acid (IFA) compliance in Ghana was high [46]. Other studies explain that this lack of knowledge affects adolescent food intake, which is not optimal [47]. Adolescents’ lack of knowledge can be corrected by continuing health education.

One of the obstacles in providing iron deficiency anemia services is that the data on IDA in adolescents in developing countries are not optimal [48]. Quality iron deficiency anemia services help the ministry of health to develop strategies for iron deficiency anemia control programs [36]. Iron supplements improve cognitive and physical ability, productivity, and well-being [1]. Thus, monitoring the quality of iron supplements is an important aspect that should not be overlooked [36].

Another obstacle in the iron deficiency anemia prevention program is the lack of motivation (adherence to the consumption of iron supplements) and health awareness. Awareness of the fact that iron and folic acid supplementation in adolescent girls can help to build iron reserves when entering married life and becoming a mother could be a solution for the problem of iron deficiency anemia in pregnant women [45]. A study by Laimi et al. stated that the problem of iron deficiency anemia was exacerbated by the lifestyle and level of awareness of adolescents surrounding iron deficiency anemia prevention [49]. Motivation with respect to the consumption of IFA tablets is influenced by adolescents’ perception that IFA is a contraceptive or fertility drug, and therefore refuse to take it [50]. This must be supported by the role of health workers in providing counseling related to how to take tablets. In addition, our study shows that adolescents think that iron supplements cause side effects such as nausea and headaches. Adolescents do not take iron supplements because they make them feel queasy and lightheaded [51,52]. The effects of nausea and dizziness due to iron supplements can be avoided if taken correctly.

Adolescents do not want to use health services for prevention; one of the main reasons that they visit medical facilities is their illness. Positively, if teens are offered health instruction in the form of interactive movies, they will be highly motivated.

Some information about adolescent health programs was not conveyed. Currently, iron deficiency anemia services are mainly focused on infants, young children, pregnant women, and breastfeeding mothers, while the focus on adolescents is still lacking [48]. Access to health services is still poor. This is due to the absence of adolescent health service facilities, including access to health information. In general, adolescents in rural areas experience malnutrition due to a lack of knowledge caused by limited access to health [53].

Health education about IDA and its prevention is still not optimal. In addition, distance and transportation costs regarding health services are obstacles to accessing health services. Access to health services is also a factor that affects IDA [1]. This includes access to different health services, such as access to health information, access to receiving iron supplements, and access to community health service centers. Giving iron supplements to school adolescents is one strategy for preventing iron deficiency anemia [36]. The existence of a mobile vehicle is expected to provide health services around adolescents’ locations of residence, so that health services can be reached easily.

Another obstacle comes from the community, who still misunderstand the provision of iron supplements [54]. Iron deficiency anemia does not only involve adolescents themselves; parents and society also play an essential role in preventing iron deficiency anemia [37,55]. Some parents play no role in the prevention of IDA, as can be seen from the interviews, where it was stated that parents only gave medicine when adolescents were sick and only gave traditional medicine. On the other hand, the role of parents in providing varied and iron-containing foods must be considered [43]. Most parents believe in herbal ingredients. This is in line with the research of Utami et al., who stated that knowledge among most parents and adolescents about iron deficiency anemia is still lacking. They also do not know the food sources that might prevent iron deficiency anemia, and they eat according to local customs. Adolescents revealed that parents’ opinions about iron deficiency anemia influenced their decision to consume iron supplements. Guidelines for proper food intake must accompany the prevention of iron deficiency anemia [44]. Several other studies have reported that it is essential to involve the community in increasing adolescent compliance in consuming iron supplements [51].

In addition to support from parents/family, most of the adolescents mentioned that they received information about iron deficiency anemia and its prevention from teachers at school. This is supported by previous research, which states that the role of teachers in efforts to reduce the incidence of iron deficiency anemia is very important, because teachers can transfer knowledge to their students [42]. In addition to parental support, teacher support is also important. Several respondents stated that the teacher’s role was not optimal, due to the teacher’s limited time. In fact, by empowering teachers, the knowledge and motivation of adolescents to prevent iron deficiency anemia could be increased. This is supported by research conducted in Crete, which concluded that providing information to teachers, adolescents, and parents is a triangulation strategy for overcoming nutritional problems, especially iron deficiency anemia [56]. Teachers have an important role in increasing knowledge related to iron deficiency anemia [57]. The teacher hopes that there will be material about health that is included in subjects at school through health promotion media. Adding nutritional education to schools is one suggested strategy, as it can change habits, including eating habits [56]. 

School-based intervention through health education regarding the causes of iron deficiency anemia is very important as an effort for reducing the number of iron deficiency anemia sufferers among adolescents [48]. Regarding facilities, several adolescents said that there was no posyandu for adolescents in their neighborhood, only posyandu for pregnant women and toddlers. Support from the community is needed to provide posyandu facilities so that iron deficiency anemia prevention services can reach all adolescents, both those who are in school and those who are not. The themes identified in this study serve as a model for an integrated healthcare system in which all relevant stakeholders are required to be successful in preventing and treating iron deficiency anemia in adolescent girls.

## 5. Conclusions

The model of an integrated health care system for preventing iron deficiency anemia in adolescent girls consists of several essential aspects, namely policymakers’ commitment, governance, and quality; adolescent lifestyles; adolescent self-factors; access to health services; and parent, teacher, and community support. However, the findings of this study can serve as a reference for other developing countries in preventing iron deficiency anemia in adolescent girls. For successful programs, everyone—not only the health industry, teenagers, and the affected parties—must collaborate.

## Figures and Tables

**Table 1:** Sociodemographic characteristics of the participants.

Characteristic	*n* = 41	%
Adolescent Girls (Aged 16–18 years)	12	29.4
School Program - High school (SMA) - Vocational and pre-professional high school (SMK) - Madrasah Aliyah (MA/Islamic schooling equivalent of SMA)	4 4 4	9.8 9.8 9.8
Parents (Aged 36–68 years)	6	14.7
Education - Elementary School (SD) - Junior High School (SMP) - High School (SMA)	2 2 2	4.9 4.9 4.9
Occupation - Self-employment - Housewife	3 3	7.3 7.3
Teachers (Aged 26–54 years)	16	38.9
Education - Bachelor’s Degree - Master’s Degree	13 3	31.6 7.3
Health Workers (Aged 22–50 years)	4	9.8
Education - Associate Degree - Bachelor Degree	2 2	4.9 4.9
Policymaker (Aged 38–51 years)	3	7.2
Education - Associate Degree - Bachelor’s Degree - Master’s Degree	1 1 1	2.4 2.4 2.4

**Table 2:** Analytical framework.

No	Theme (Construct)	Category
1	The commitment of stakeholders (Puskesmas, Health Office, Education Office, and Ministry of Religion)	1. Adolescent counseling room 2. Availability of infrastructure 3. Availability of financial support 4. Quality of health workers 5. Health worker behavior 6. Availability of iron deficiency anemia health workers
2	Governance	1. Evaluation monitoring 2. Assignment 3. Coordination 4. Standard operating procedures
3	Adolescent Lifestyle	1. Knowledge 2. Healthy lifestyle
4	Quality	1. Quality services 2. Quality of iron supplements
5	Adolescent Self-Factors	1. Trust 2. Motivation
6	Health Services Access	1. Access to health information 2. Access to community health services 3. Access to iron supplements
7	Social Support	1. Parent/family support 2. Teacher/school support 3. Social support

## Data Availability

Not applicable.
